# Neuroleptic malignant syndrome in an adolescent with CYP2D6 deficiency

**DOI:** 10.1007/s00431-013-2208-z

**Published:** 2013-11-20

**Authors:** Agnieszka Butwicka, Szymańska Krystyna, Włodzimierz Retka, Tomasz Wolańczyk

**Affiliations:** 1 Department of Medical Epidemiology and Biostatistics, Karolinska Institutet, PO Box 281, 171 77 Stockholm, Sweden; 2 Department of Child Psychiatry, Medical University of Warsaw, 24 Marszałkowska St., 00-575 Warsaw, Poland; 3 Department of Paediatric Anaesthesia, Medical University of Warsaw, Warsaw, Poland

**Keywords:** Adverse drug reactions, Pharmacogenetics, CYP2D6, Poor metabolizer, Neuroleptic malignant syndrome

## Abstract

We describe a patient with dystonia and psychotic symptoms treated with standard doses of antipsychotics, who developed neuroleptic malignant syndrome (NMS). A 16-year-old male with a history of misuse of dextromethorphan and pseudoephedrine for recreational purpose presented with dystonia and a psychotic episode. Following continuous treatment with olanzapine (10 mg/day), repeated injections of levomepromazine (37.5 mg/day), and a single injection of haloperidol (2.5 mg), the patient developed NMS. Muscular rigidity, fever (up to 41 °C), hypotension (100/70 mmHg), tachycardia (120 beats per minute), tachypnea (26 breaths per minute), elevated leukocyte count (up to 16.6 × 10^3^/μL), and elevated serum creatinine phosphokinase (CPK) (up to 15,255 U/L) were observed. A diagnosis of NMS was made according to the DSM-IV TR criteria. Genotyping revealed that he was homozygous for a non-functional CYP2D6*4 allele. The case highlights the importance of therapeutic drug monitoring in identification and differentiation of drug-induced effects in psychiatric disorder to prevent NMS and its complications. In addition, genotyping of CYP2D6 might be considered in patients with symptoms suggestive of drug toxicity who are treated with neuroleptics metabolized via the CYP2D6 pathway, as carriage of one or more non-functional alleles may increase the risk for adverse reactions, such as NMS.

## Introduction

The use of atypical antipsychotic has increased rapidly in recent years in pediatric populations worldwide [1]. Neuroleptic malignant syndrome (NMS) is a rare but potentially fatal complication of treatment with antipsychotics. Clinical features of NMS include muscle rigidity, hyperpyrexia, autonomic instability, mental status changes, and evidence of muscle catabolism [2]. One of the hypotheses regarding its etiology postulates that susceptibility to NMS may be associated with variation in genes that code for metabolic enzymes [7]. We report on a pediatric patient who developed NMS while being treated with standard doses of antipsychotics due to an acute psychotic episode. Clinical observation suggested drug toxicity due to impaired drug metabolism. Genotyping of the *CYP2D6* gene confirmed the carriage of two dysfunctional alleles.

## Case report

In September 2009, a 16-year-old male (weight: 65.5 kg, height: 1.83 m, BMI = 19.6 kg/m^2^) was admitted to a pediatric neurology unit due to dystonia. A month before, the patient became withdrawn and developed insomnia and twisting movements of the left extremities. These symptoms began after a 1-month period of using medication containing dextromethorphan and pseudoephedrine for recreational purpose. Upon admission to the pediatric neurology unit, he was well oriented and followed instructions. Neurological examination showed no alteration except for sustained twisting of the left extremities. Results of laboratory tests, electroencephalography (EEG), and computed tomography of the head and neck were normal. During hospitalization his mental status deteriorated. He became confused and illogical. The consulting psychiatrist diagnosed a psychotic episode and transferred the patient to the adolescent inpatient psychiatric unit.

**Fig. 1:**
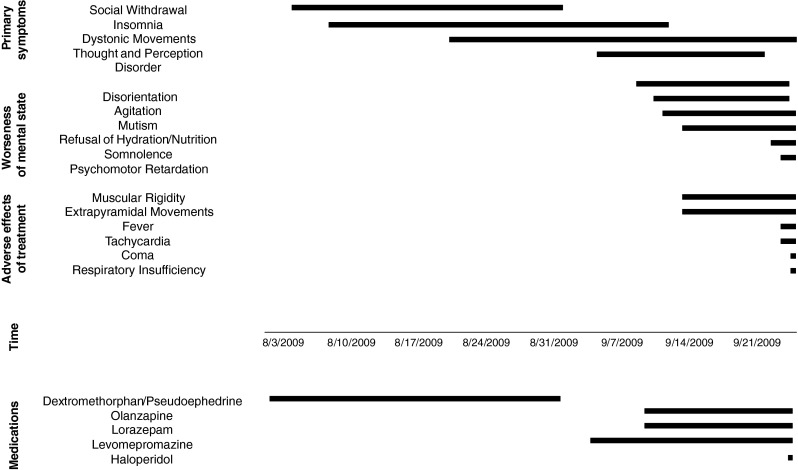
Timeline of presented symptoms and administered medication prior to admission to the intensive care unit

At the time of this transfer, the patient was awake and revealed delusional thoughts and hallucinations. Treatment with orally administered olanzapine (10 mg/day) and lorazepam (as required) with intramuscular injections of first-generation antipsychotic medication—levomepromazine (25 mg/day)—was initiated. The next day, levomepromazine was increased to 37.5 mg/day. During the course of treatment, dystonic movement worsened. Opisthotonic trunk extension, intermittent, sustained extension movements of the arms, and incomprehensible screaming were observed. The patient had no verbal communication with the medical staff, was agitated, unable to sleep, and refused to eat and drink. Intravenous fluids were administered for dehydration. In 2 weeks of treatment, he became drowsy with decreased physical activity. Due to recurrent episodes of agitation, the patient received 1–3 mg/day of lorazepam. An episode of aggression towards medical staff required one intramuscular injection with haloperidol (2.5 mg). The timeline of presented symptoms and administered medications is displayed in Fig. 1. At the 16th day of treatment with antipsychotics, the patient was transferred to the University Child Psychiatry Department.

At the time of this transfer, the patient was unconscious and showed muscular rigidity and extrapyramidal symptoms. Fever (up to 41 °C), hypotension (100/70 mmHg), tachycardia (120 beats per minute), and tachypnea (26 breaths per minute) were noted. Laboratory analysis revealed a leukocyte count (WBC) of 13.40 × 10^3^/μL, serum creatinine phosphokinase (CPK) level of 639 U/L, and normal liver and renal function tests.

On the following day, the patient became diaphoretic, with a Glasgow Coma Scale of 7 points (range 3–15 points) indicating a state of coma. CPK increased to 2,458 U/L, WBC to 16.6 × 10^3^/μL, creatinine (SCC) to 1.70 mg/dL, and blood urea nitrogen (BUN) to 79.0 mg/dL. The patient was transferred to an intensive care unit (ICU), intubated and mechanically ventilated because of respiratory insufficiency. Generalized tonic–clonic seizures were observed. Acyclovir was administered for potential herpes encephalitis and dantrolene to counter potential NMS. Electrocardiogram revealed elevation of the ST segment with T wave inversion. Elevated troponin levels and myoglobin detected in urine suggested myocardial injury. An echocardiogram showed left ventricular hypokinesis. Serum CPK levels continued to rise over the following 6 days and reached a peak of 15,255 U/L. BUN and SCC also increased for 3 days, up to 79.6 and 2.1 mg/dL, respectively. Organic pathology of the brain was ruled out after repeatedly normal magnetic resonance images and cerebrospinal fluid examination (glucose concentration 70.0 mg/dL, protein concentration 34 mg/dL, and an absence of cells). Polymerase chain reaction (PCR) ruled out herpes simplex virus infection, and acyclovir was discontinued. Six weeks after the last administration of neuroleptics, urine toxicology was done. The presence of haloperidol was detected, but not olanzapine. The presence of levomepromazine was not measured. There was no test performed to measure any plasma drug concentration. Real-time PCR showed that the genotype of CYP2D6 was *4/*4. These findings indicated lack of CYP2D6 activity.

As other conditions were excluded, the diagnosis of NMS was made based on symptoms of muscle rigidity, elevated temperature, and leukocytosis. After 10 weeks of hospitalization in the ICU, the patient was readmitted to the psychiatric unit. His weight had decreased to 41 kg (BMI = 12.2 kg/m^2^). At the time of readmission, he was agitated and visually hallucinating. Extrapyramidal symptoms were present. Disturbance of mental status fluctuated during the course of the day with worseness of agitation in the nights. The diagnosis of delirium was stated. Secondary generalized tonic–clonic seizures were noted every second day. Agitation was treated with ziprasidone, and gabapentin was introduced for seizures. During the subsequent 6-month hospitalization, the patient's condition gradually improved, and he gained 12.5 kg (weight: 53.5 kg, height: 1.83 m, BMI = 16.0 kg/m^2^). In June 2010, he was discharged from inpatient center with diagnosis of schizophreniform disorder complicated by neuroleptic malignant syndrome according to DSM-IV TR criteria. He remained under regular review in the outpatient psychiatric clinic. Control EEGs performed 6 and 12 weeks after the above episode were normal. Ziprasidone and gabapentin were gradually withdrawn, and no other medications were required. In September 2010, he went back to school. However, due to learning difficulties, he had to repeat the class. After 2.5 years of outpatient follow-up, he still shows persistent mild memory and concentration impairment and emotional lability. There has been no further evidence of psychotic symptoms or seizures.

## Discussion

This is the first report of a pediatric patient with NMS and confirmed CYP2D6 deficiency. Symptoms presented by the patients such as fever, rigidity, tachycardia, leukocytosis, diaphoresis, abnormal blood pressure, tachypnea, altered mental status, and elevated CK level are consistent with clinical presentations of other patients with NMS [11]. Remarkably, the development of NMS was preceded by catatonic symptoms such as mutism, refusal of food and fluid intake, and decreased and excesive motor activity. There are reports suggesting that neuroleptic-induced catationa may be a stage progressing to NMS [9]. In the presented case, catatonic symptoms occurred after introduction of neutroleptics, and further treatment even worsen the mental state of the patient (Fig. 1).

This case illustrates a multifactorial cause of NMS. The primary risk factors are probable disproportionally high drug concentrations - which were unfortunetely not measured - and toxicity resulting from repeated administration of oral and intramuscular medication in a patient with dysfunctional metabolic pathways. This patient received repeated daily doses of olanzapine. The role of olanzapine and its combination with levomepromazine in the development of NMS has been previously described [5, 8]. The plasma level of olanzapine should not have exceed therapeutic levels as it is mainly metabolized by CYP1A2 and to a lesser extent by CYP2D6. However, the clinical presentation, with the lack of improvement and sedation on adequate doses of medications combined with worseness of catatonia, suggests drug toxicity. Metabolism of levomepromazine and haloperidol is catalyzed by CYP2D6 and if given to the patient with lack activity of this enzyme might have induced adverse effects even on standard doses [3]. Furthermore, nonfunctional alleles of CYP2D6 have been suggested to affect vulnerability to NMS in Japanese population [6]. Other factors such as dehydration, malnutrition, and severe clinical presentation with agitation may also contribute to the development of NMS. Initial symptoms of the patient might be also related to the abuse of dextromethorphan- and pseudoepedrine-containing remedies as both medications are metabolized by CYP2D6 and known to cause dystonia and psychotic symptoms [10].

Our report highlights the need for therapeutic drug monitoring (TDM) [4]. TDM is economically reasonable and can detect different pharmacokinetic problems, not limited to CYP polymorphisms. Unfortunately, in Poland, TDM of neuroleptics was not implemented in the clinical routine and even if recommended remains unavailable in psychiatric centers. Described patient had several indications for measuring plasma concentration of medication such as possible medication-related onset of symptoms, adverse effects on standard doses of medication, suspected impaired drug metabolism, and being an adolescent patient. Plasma levels of drugs involved in this case would have been helpful to evaluate the origin of symptoms, modify the treatment, and avoid dramatic complication.

## Abbreviations

BUN: Blood urea nitrogen
CPK: Serum creatinine phosphokinase
DSM-IV TR: Diagnostic and Statistical Manual of Mental Disorders, Fourth Edition, Text Revision
EEG: Electroencephalography
ICU: Intensive care unit
NMS: Neuroleptic malignant syndrome
PCR: Polymerase chain reaction
SCC: Creatinine
TDM: Therapeutic drug monitoring
WBC: Leukocyte count
